# Patterning a hydrogen-bonded molecular monolayer with a hand-controlled scanning probe microscope

**DOI:** 10.3762/bjnano.5.203

**Published:** 2014-10-31

**Authors:** Matthew F B Green, Taner Esat, Christian Wagner, Philipp Leinen, Alexander Grötsch, F Stefan Tautz, Ruslan Temirov

**Affiliations:** 1 Peter Grünberg Institut (PGI-3), Forschungszentrum Jülich, 52425 Jülich, Germany; 2 Jülich Aachen Research Alliance (JARA)-Fundamentals of Future Information Technology, 52425 Jülich, Germany; 3 Leiden Institute of Physics, Universiteit Leiden, Niels Bohrweg 2, 2333 CA Leiden, The Netherlands; 4 Unit Human Factors, Ergonomics, Federal Institute for Occupational Safety and Health (BAuA), 44149 Dortmund, Germany

**Keywords:** atomic force microscopy (AFM), scanning tunneling microscopy (STM), single-molecule manipulation, 3,4,9,10-perylene tetracarboxylic acid dianhydride (PTCDA)

## Abstract

One of the paramount goals in nanotechnology is molecular-scale functional design, which includes arranging molecules into complex structures at will. The first steps towards this goal were made through the invention of the scanning probe microscope (SPM), which put single-atom and single-molecule manipulation into practice for the first time. Extending the controlled manipulation to larger molecules is expected to multiply the potential of engineered nanostructures. Here we report an enhancement of the SPM technique that makes the manipulation of large molecular adsorbates much more effective. By using a commercial motion tracking system, we couple the movements of an operator's hand to the sub-angstrom precise positioning of an SPM tip. Literally moving the tip by hand we write a nanoscale structure in a monolayer of large molecules, thereby showing that our method allows for the successful execution of complex manipulation protocols even when the potential energy surface that governs the interaction behaviour of the manipulated nanoscale object(s) is largely unknown.

## Introduction

The scanning probe microscope (SPM) is an excellent tool for the manipulation of atoms and molecules on surfaces due to its high spatial imaging resolution and atomic-scale precision [1–7]. Today, controlled SPM manipulation of individual atoms and small molecules is a routine operation [6–8]. It has been recognised that the outcome of such manipulations is fully defined by the microscopic interactions between the manipulated atom or molecule, the surface and the tip [5].

If the manipulated object is an individual atom or a small molecule its internal degrees of freedom can be neglected (as for a point-like particle) such that the state of the particle is fully described by its three spatial coordinates. Since the position of the tip apex is also defined by a set of three coordinates, the full state space of an SPM junction that contains one point-like particle essentially has at least six independent dimensions [9]. Therefore in order to perform a successful SPM manipulation one ideally needs to know the junction potential function defined over the whole 6-D state space. Because most of the detailed studies of SPM manipulation have been performed on individual atoms or small molecules adsorbed on surfaces with a highly symmetric structure, their success can be explained to a large extent by the fact that the high symmetry of the surface considerably simplifies the potential of the junction in multifunctional state space [5,8]. At the same time it is clear that the realisation of more advanced nanoscale functions will eventually rely on highly controlled manipulations with molecular objects of larger size, possessing numerous internal degrees of freedom and adsorbed on surfaces with a more complex and thus less symmetric structure.

Unfortunately, the behaviour of large molecules on surfaces is generally not well understood. Despite the fact that studies of complex molecular adsorption are progressing quickly, even in the best-studied model cases a full and quantitatively precise picture of the molecular adsorption potential (even in the absence of the SPM tip) is not yet available. For systems that contain a larger number of molecules that may simultaneously interact with the surface, the SPM tip and each other, reconstruction of the potential does not seem realistic in the nearest future.

How can we nevertheless manipulate large molecules successfully, despite lacking full knowledge of their complex interaction potential? Generally, the manipulation act is defined as a trajectory that connects the initial and the final states of the junction in its multidimensional state space. In SPM such trajectories can only be executed by controlled changes of the spatial coordinates of the tip. The other degrees of freedom of the junction, namely the centre of mass and the internal degrees of freedom of the manipulated molecule, cannot be directly controlled; instead they relax spontaneously as the tip is moved along its 3-D trajectory. Their relaxations are always directed such that they minimize (locally) the total potential of the junction. For a manipulation to be "successful" the sequence of spontaneous relaxations of molecular degrees of freedom must steer the junction into the final state of the manipulation. If the potential of the system were known at each point of its state space, the identification of the desired tip trajectory would become a mathematical problem. In reality, since the potential is not known “successful” trajectories can only be determined with the help of an experiment in which the relevant regions of the potential landscape are explored in a “trial and error” fashion and the obtained information is finally used for learning. In future one could envision a computer-driven SPM that automatically learns successful manipulation protocols through performing specific experiments on single molecules and analysing their outcomes. Here we demonstrate the principal possibility of such learning by substituting a computer-driven system with a human operator controlling the position of the SPM tip with their hand. Our experiments directly show that the operator efficiently finds trajectories for the intentional manipulation of large organic adsorbates without prior knowledge of the potential to which the manipulated system is subjected.

## Experimental

**Figure 1:**
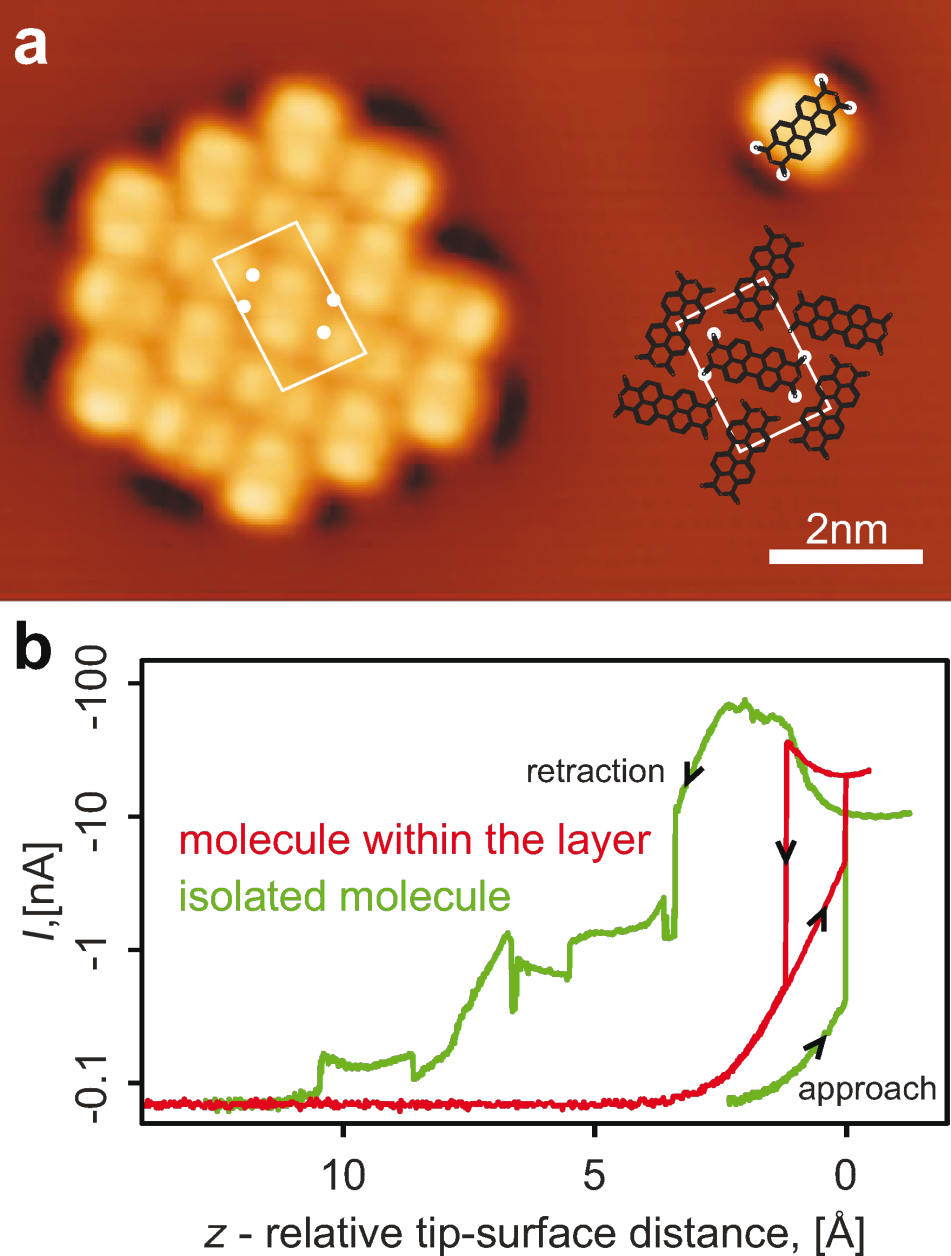
(a) 13 × 8 nm^2^ STM image of a PTCDA island grown on an Ag(111) surface and of an isolated PTCDA molecule detached from it. The white rectangle marks the unit cell of the monolayer. The structure of the PTCDA/Ag(111) layer is displayed on the right. The positions of the carboxylic oxygen atoms of PTCDA are marked by white circles. All of the STM images were post-processed with WSxM software [14]. (b) *I*(*z*) curves measured upon tip approach and subsequent retraction executed over one of the carboxylic oxygen atoms of PTCDA with the applied bias voltage of *V* = −5 mV. Black arrows superimposed on the red and green curves show the direction of the tip movement. The contact event is observed as a sharp increase of *I*(*z*). The isolated PTCDA molecule can be pulled away from the surface simply by retracting the tip vertically (green curve). PTCDA molecules that reside inside monolayer islands resist pulling, which breaks their contact to the tip prematurely (red curve). The relative tip–surface distance scale (*z*) was aligned such that the contact point defined its zero value.

For the demonstration of our manipulation technique we chose one of the best-studied cases of the adsorption of complex organic molecules: the well-ordered interface formed by the archetypal organic semiconductor 3,4,9,10-perylene tetracarboxylic acid dianhydride (PTCDA) on a single-crystalline Ag(111) surface [10] (see Figure 1a). An Ag(111) single crystal was cleaned by repeated Ar-sputtering and annealing cycles. A small coverage of PTCDA molecules (less than 10% of a monolayer) was subsequently deposited from a custom-built Knudsen-cell onto the freshly prepared Ag(111) surface kept at room temperature. Immediately after deposition the sample was moved into the microscope and cooled to 5 K. Prior to the imaging and manipulation experiments the SPM tips were prepared by voltage pulses of 3–6 V (applied to the sample) and by crashing 10–30 Å deep into the clean Ag(111) surface whilst simultaneously applying a voltage of 0.1–1 V. The cleanness of the tip was validated by STM imaging of the former lowest unoccupied molecular orbital (LUMO) of PTCDA [10] and spectroscopy of the Ag(111) surface state. All PTCDA images shown were made with STM at *I* = 0.1 nA and with an applied bias voltage of *V* = −0.34 V that facilitates the intramolecular resolution corresponding to the LUMO. All of the reported experiments were performed in situ under ultra high vacuum conditions.

The adsorption mechanics of PTCDA on Ag(111) is well understood: a PTCDA molecule binds to the metal surface through an extended bond that involves charge transfer into its LUMO and also locally with its four carboxylic oxygen atoms [10–11] (marked by white circles in Figure 1a). The same atoms enable SPM manipulation with the molecule, since an individual carboxylic oxygen atom can bind the molecule to the SPM tip [12]. For a molecule residing within a compactly ordered monolayer, the same carboxylic groups are involved in hydrogen-bonds with the C–H side groups of neighbouring PTCDA molecules [10–11]. These intermolecular interactions bind the molecules to each other, holding them tightly within the molecular islands [13].

An attempt to manipulate PTCDA thus faces a conspicuous practical problem: while an isolated molecule that has no neighbours can be contacted and lifted from the surface with the SPM tip in a straightforward manner, the interactions between the molecules foil most of the attempts to remove a molecule residing inside the compact molecular monolayer [15–16] (see Figure 1b). Although the nature of the forces that hold the layer together is qualitatively understood, due to the lack of quantitative information it is not clear a priori how to remove a molecule from the layer with the SPM tip; because of the prohibitively large state space it would also be impossible to calculate this with reasonable effort. As will be shown here, hand-controlled manipulation (HCM) using the SPM tip allows us to find a manipulation protocol that removes single PTCDA molecules from the molecular monolayer very reliably.

We performed the molecular manipulation with a commercially available SPM. Our instrument, the low-temperature combined non-contact atomic force/scanning tunnelling microscope (NC-AFM/STM) from CREATEC, allows for a stable and precise positioning of the tip, while simultaneously measuring the current flowing through the junction (*I*) and the frequency shift of the oscillating tip (Δ*f*). Measuring Δ*f* provides additional information about the microscopic junction structure [15–16]. For the AFM functionality we used a qPlus sensor [17] manufactured by CREATEC. The AFM/STM tip was made from a 0.3 mm long and 15 μm thick PtIr wire glued to the tuning fork of the qPlus sensor, and sharpened with a gallium focused ion beam (FIB). The resulting resonance frequency of the qPlus sensor was *f*_0_ = 30,300 Hz with a quality factor of *Q* ≈ 70,000. Contacting and manipulation were performed with the qPlus sensor oscillating with an amplitude of *A*_0_ ≈ 0.2–0.3 Å. Interactions in the junction were monitored by measuring the frequency shift Δ*f*(*z*) ≈ −(*f*_0_/2*k*_0_)*dF_z_*/*dz*, where *k*_0_ = 1800 N/m is the stiffness of the quartz tuning fork used.

**Figure 2:**
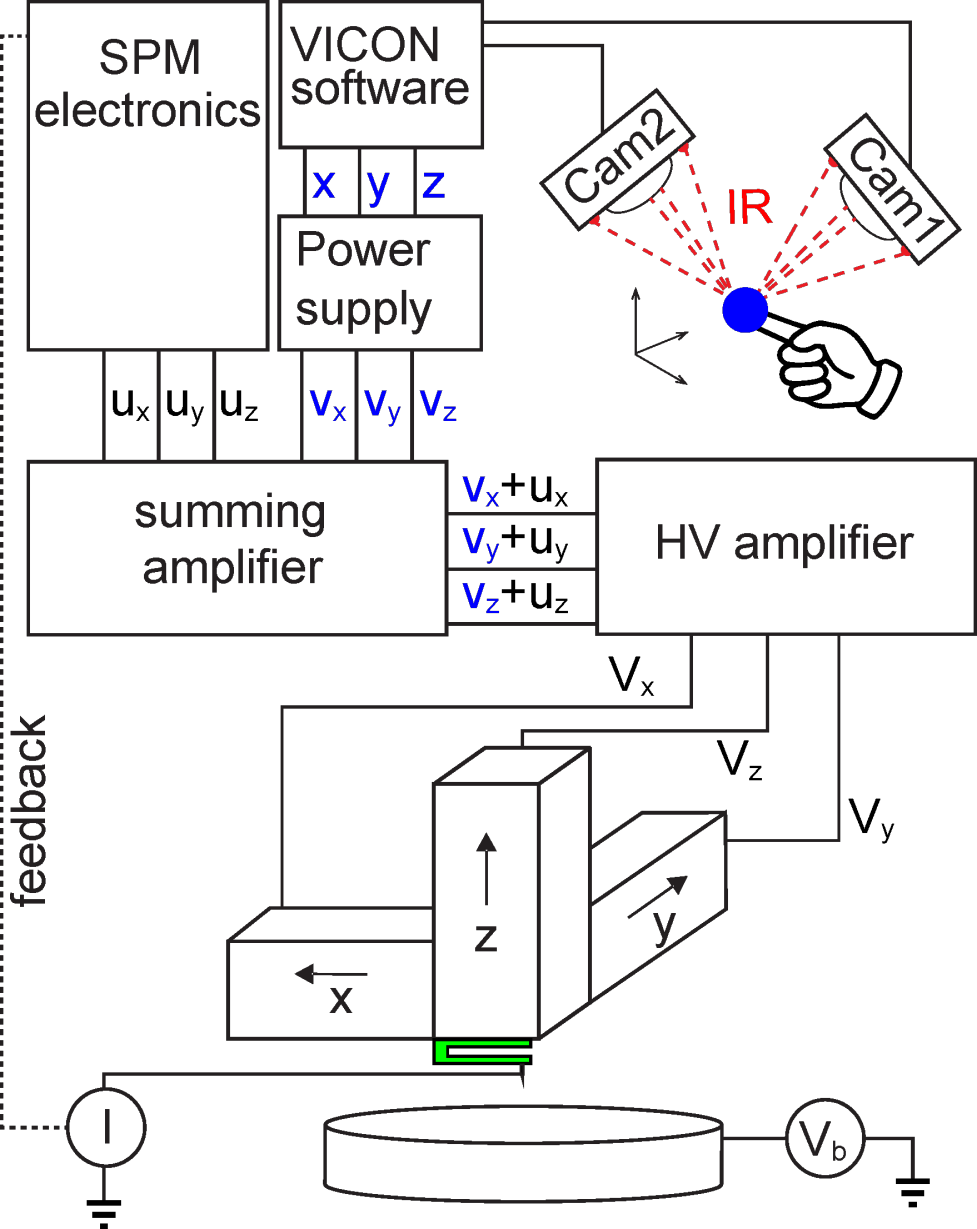
Scheme of the set-up for manual control of the SPM tip. Lamps mounted on the front of the two cameras emit infrared light that is reflected by a single marker fixed rigidly to the hand of the operator. The reflected light is captured by the cameras; with two cameras full three-dimensional triangulation is achieved. At the system output the real-time *x*(*t*), *y*(*t*), *z*(*t*)-coordinates of the marker are extracted. These coordinates are converted into a set of three voltages *v_x_*, *v_y_*, *v_z_* that are further added to the *u_x_*, *u_y_*, *u_z_* voltages of the SPM software used to control the scanning piezo-elements of the microscope. In this way when the feedback loop is closed the position of the SPM tip is controlled by the SPM software, but when the feedback loop is open the tip is controlled by the hand of the operator. During the manipulation *v_x_* + *u_x_*, *v_y_* + *u_y_* and *v_z_* + *u_z_* voltages are sampled at a frequency of 1 kHz.

The essence of our approach lies in the coupling of the sub-angstrom precise positioning of the tip of our instrument to the motion of the operator's hand [18]. This is achieved with the help of a commercial motion tracking system from VICON (see Figure 2). The VICON software was used to obtain Cartesian coordinates of a marker attached to the hand of the operator and feed them into a high precision power supply from STAHL ELECTRONICS that generated three voltages, *v_x_*, *v_y_* and *v_z_*, which were added to the voltages *u_x_*, *u_y_*, *u_z_* used by the scanning probe software to control the position of the SPM tip. The system was calibrated such that 5 cm of hand motion corresponded to 1 Å of tip movement, and calibration constants were chosen to be the same for *x*, *y*, and *z* directions. The tip manipulation speed did not exceed 0.2 Å/s. This limitation was imposed by the latency time of the communication channel between the tracking software and the power supply generating the voltages *v_x_*, *v_y_*, *v_z_* (see Figure 2). The spatial uncertainty introduced by the motion tracking software was equal to 0.01 Å along each of the axes (*x*, *y*, *z*). The uncertainty introduced by the electrical noise in the low- and high-voltage amplifiers was about 0.01 Å along *z* and 0.05 Å along *x* and *y* directions. The coupling latency time was 50 ms. The contacting and molecular manipulation was performed at *V*_b_ = −5 mV. In total 48 molecules were extracted from the monolayer. Each HCM was preceded by an attempt to lift the molecule by moving the tip straight up from the surface; only five molecules were removed in this manner.

Each individual HCM started by acquiring a constant current STM image of the molecule to be manipulated. The tip was then parked over the carboxylic oxygen atom of PTCDA that had been selected for contacting and the current feedback loop of the SPM software was opened. The contact to the molecule was established by approaching the tip vertically towards the surface; this approach was effected by downward movement of the hand of the operator. Over the course of HCM the current *I* flowing through the junction and the frequency shift Δ*f* were displayed on the screen of an oscilloscope and served as feedback signals for the operator. Formation (loss) of the contact was monitored in real time by a sharp increase (decrease) of *I* (cf. Figure 1b) or a kink in Δ*f* [15–16]. After establishing the contact between the tip and the molecule, the operator retracted the tip along an arbitrary three-dimensional trajectory. If the contact to the molecule was lost prematurely, the tip was moved back to the initial parking position by zeroing the *v_x_*, *v_y_* and *v_z_* voltages and the manipulation was re-initiated. If contact was maintained up to retraction distances of 10–15 Å, the tip was moved, with the help of the SPM software, laterally at constant height to a clean silver surface area. There an attempt to re-deposit the molecule from the tip back to the surface was made. Re-deposition was performed by approaching the tip with the removed PTCDA molecule hanging on its apex towards the Ag(111) surface and applying a voltage pulse of 0.6–1 V. Afterwards the current feedback loop was closed and the manipulation area was scanned in constant current STM mode (a movie that was made of the scanned STM images can be found in the Supporting Information). If the state of the tip apex was changed during HCM it was reshaped by gentle dipping into the surface.

With this approach and without any prior experience it took about 40 minutes to remove the first molecule from the layer. Repeating the experiment, we observed that the average time necessary to remove one molecule decreased to 13 minutes after about 10 successful attempts. We stress here that this learning was based entirely on rather sparse information about the junction, namely the conductance at a fixed bias voltage and the frequency shift Δ*f* related to the *z*-gradient of the vertical force [15–16].

## Results and Discussion

**Figure 3:**
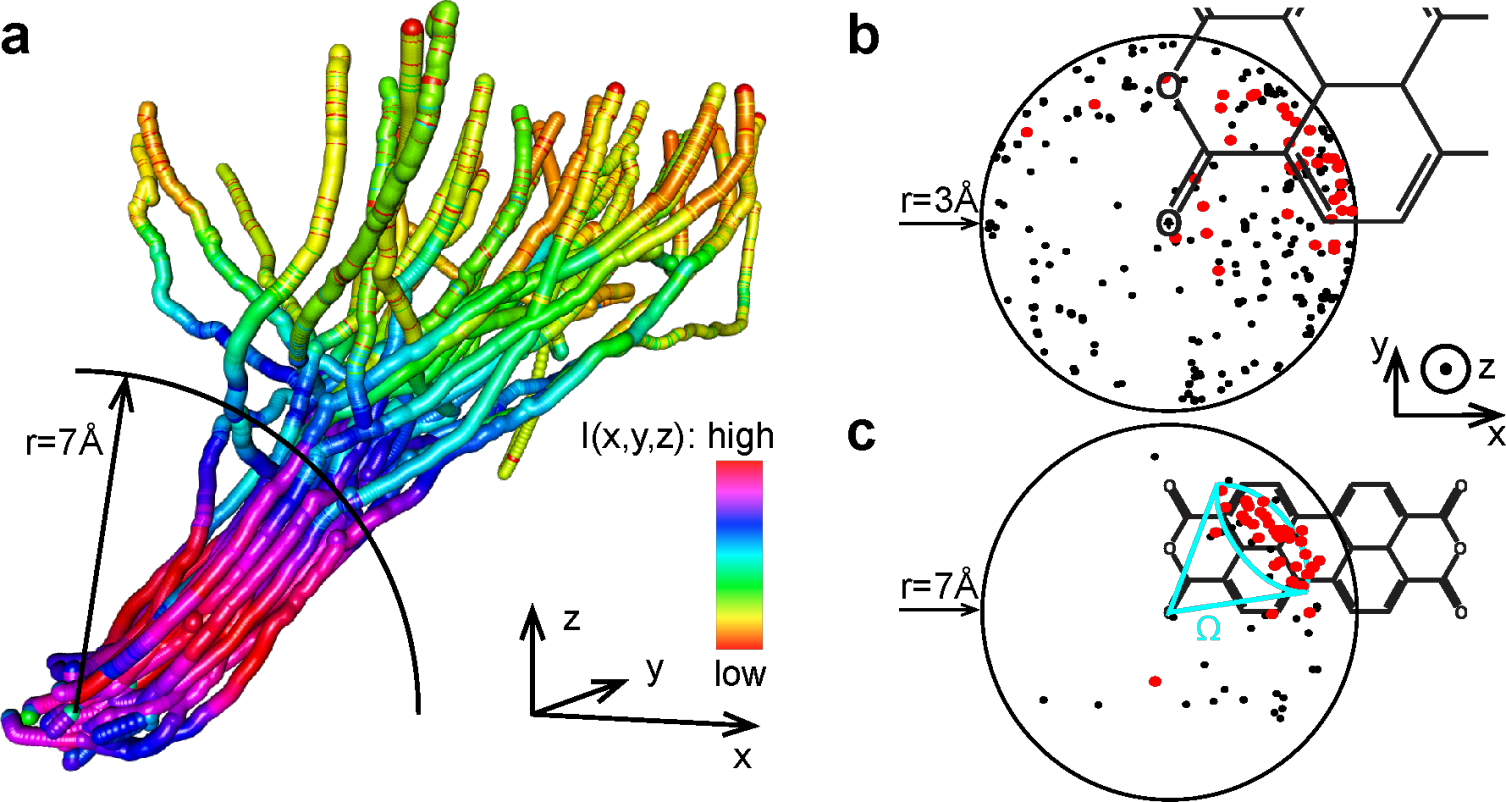
a) A perspective view on a set of 34 3-D manipulation trajectories that resulted in the removal of PTCDA molecules from the monolayer. In order to facilitate plotting, the density of recorded data was reduced by a factor of 100 to a sampling frequency of 10 Hz. Each point of the trajectory is plotted as a sphere with a radius of 0.2 Å, corresponding to the amplitude of the oscillations of the AFM/STM tip. The colour of the sphere reflects the value of *I*(*x*, *y*, *z*) measured at the given point of the manipulation trajectory. The black circle shows the boundary of the sphere from Figure 3c. For a more detailed view of the displayed 3D trajectories download the 3D animation or the interactive 3D model from Supporting Information. b,c) Full statistics of manipulation trajectories (including unsuccessful ones) (top view). The circle marks the boundary of a sphere with the radius 3 Å (b) and 7 Å (c) the center of which was placed at the position of the carboxylic oxygen atom through which the molecule was contacted by the tip. Red (black) points mark locations where the successful (unsuccessful) trajectories penetrate the sphere. Bunching of the successful trajectories in a narrow solid angle is visible at larger tip–surface distances.

Inspecting Figure 3a, which displays the 3-D trajectories that successfully extracted the PTCDA molecules from the layer, we note several interesting observations. First we see that all of the successful trajectories tend to “bunch” in a relatively narrow solid angle. The correct determination of that angle thus largely defines the success of the manipulation. Here the operator determines the required solid angle by using the fact that unsuccessful trajectories terminate prematurely with the tip-molecule bond rupture. As Figure 3b shows, many of the trajectories “survive” the first 3 Å of pulling, although the ones that are going to become successful start to concentrate in the upper right quadrant. As the tip moves further away from the surface many unsuccessful traces get terminated due to the premature breaking of the tip–molecule contact. Indeed Figure 3c shows that at a distance of 7 Å most of the successful trajectories lie within the solid angle Ω (cf. Figure 3c), the direction of which suggests that the molecule is peeled off the surface starting from the corner at which the contact to the tip was established [19]. We remarked previously that the effectiveness of peeling stems from the fact that it promotes gradual (vs simultaneous) cleavage of the existing molecule–surface bonds [12,16]. In contrast to the case of an isolated molecule, when the molecule is peeled out of the compact layer the intermolecular bonds also need to be cleaved. Therefore extraction of the molecule from the layer needs a much more carefully chosen trajectory which “schedules” the cleavage of the molecule–surface bonds as well as the bonds between molecules in such a manner that the total force acting on the tip–molecule bond is kept under a critical threshold. The identification of such trajectories is performed here by the operator carrying out HCM and we find that the success of the peeling is largely defined by the direction along which the tip is moved for the first 7 Å.

Notably, after reaching a retraction distance of about 7 Å the trajectories shown in Figure 3a start to diverge from each other. This suggests that the majority of the bonds that hold the molecule within the monolayer have been cleaved by that point, thus reducing the importance of the shape of the trajectory substantially. Interestingly, the process of gradual bond cleavage is also reflected by the initial increase in the current *I*(*x*, *y*, *z*) flowing through the junction (cf. the red sections of the successful trajectories in Figure 3a). This observation is in agreement with previously published data that relate the increase of conductance through the tip–PTCDA–Ag(111) junction with the effects of de-population and de-hybridization of the LUMO of PTCDA, which occur upon the gradual breaking of the PTCDA–Ag(111) bonds [12,20–21].

**Figure 4:**
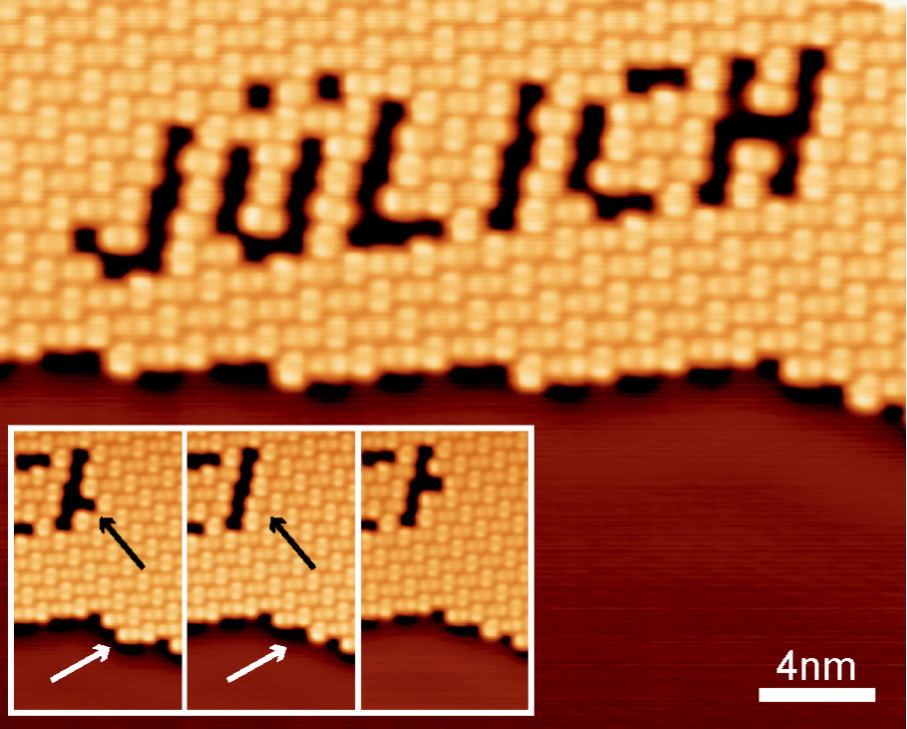
Constant current STM image of a structure consisting of 47 vacancies that were created by removing individual PTCDA molecules from the PTCDA/Ag(111) monolayer. The sequence of intermediate steps recorded during writing can be downloaded from the supplement. The three insets show the “repair” of a vacancy created by mistake. The black arrow marks the position of the error vacancy. The white arrow marks the position of the molecule at the edge of the molecular monolayer island that was used to fill the error vacancy. The molecule from the edge was removed by using the same manipulation protocol as for all other vacancies and was then placed into the error vacancy by approaching the tip to the vacancy and increasing the voltage steadily to 0.6 V.

Finally, to illustrate the reliability of the HCM, we present a structure “stencilled” into PTCDA/Ag(111) by sequentially removing single molecules from the layer (Figure 4). Importantly, the images report the very first attempt, with no previous experience and without training. A movie, assembled from constant current STM images scanned after each removal step, can be downloaded as Supporting Information. It shows that 48 molecules were extracted from the layer in a sequence defined by the will of the operator. Remarkably, it was possible to re-deposit 40 of the removed molecules onto the clean Ag(111) surface nearby, showing that the molecules are not damaged during their extraction [22]. Therefore, as Figure 4 shows, manual manipulation can also be used to “correct” errors by filling a created vacancy with a molecule that has been extracted from a different location.

## Conclusion

In summary, HCM allows for the straightforward manipulation of single molecules of large organic adsorbates in bound assemblies. The strength of the method derives from the direct manual control of the AFM/STM tip. This allows the operator to explore the unknown potential in the state space of the manipulated system, quickly determining the manipulation trajectories that steer the system into the desired final state(s). By using HCM we were able to find the trajectories of the AFM/STM tip that break the intermolecular bonds in the molecular monolayer of PTCDA/Ag(111) and write the first ever complex structure with *large* molecules.

The HCM method reported here brings us a step closer to the possibility of building functional nanoscale molecular structures. In particular, it shows that in spite of the limited information about the junction that is accessible in real time, it is nevertheless possible to efficiently learn along which paths through the multidimensional state space with its highly complex potential molecules can be manipulated successfully. In future applications of the method, this learning could be delegated to a suitable computer algorithm. At the same time, the data collected with this method may promote a deeper understanding of interactions in complex adsorption systems and thus eventually help us to make another step towards machine-controlled molecular-scale functional design.

## Supporting Information

The paper is accompanied by a ZIP archive containing the following files: The file “Manipulation-sequence.avi” contains the sequence of intermediate images recorded during the manipulation, the final result of which is shown in Figure 4. The file “3Dmovie.avi” contains an animation exhibiting the 3-D model of the recorded manipulation trajectories shown in Figure 3 (for details cf. the caption of Figure 3). The file “3Dmodel.html” contains an interactive 3-D model of the recorded manipulation trajectories. To be viewed it must be placed in the same directory as the file “CanvasMatrix.js” (included in the ZIP archive) and opened with a browser. Use the mouse to rotate or zoom the field of view of the 3-D model.

File 1 Additional experimental data
