# Supplementary material for: Patterning a hydrogen-bonded molecular monolayer with a hand-controlled scanning probe microscope
Source: Beilstein J Nanotechnol. 2014 Oct 31;5:1926–32. doi: 10.3762/bjnano.5.203 (PMC4222388; doi:10.3762/bjnano.5.203)
Supplement: File 1 — Additional experimental data [file Beilstein_J_Nanotechnol-05-1926-s001.zip › 3D model.html]

 
RGL model


Your browser does not support the HTML5 canvas element.


You must enable Javascript to view this page properly.

  
Drag mouse to rotate model. Use mouse wheel or middle button
to zoom it.

---

  
Object written from rgl 0.93.996 by writeWebGL.
